# Development and initial validation of a data quality evaluation tool in obstetrics real-world data through HL7-FHIR interoperable Bayesian networks and expert rules

**DOI:** 10.1093/jamiaopen/ooae062

**Published:** 2024-07-27

**Authors:** João Coutinho-Almeida, Carlos Saez, Ricardo Correia, Pedro Pereira Rodrigues

**Affiliations:** CINTESIS@RISE—Centre for Health Technologies and Services Research, University of Porto, 4200-319 Porto, Portugal; MEDCIDS—Faculty of Medicine of University of Porto, 4200-319 Porto, Portugal; Health Data Science PhD Program, Faculty of Medicine of the University of Porto, 4200-319 Porto, Portugal; Instituto Universitario de Aplicaciones de las Tecnologías de la Información y de las Comunicaciones Avanzadas, Universitat Politècnica de València, 46022 Valencia, Spain; CINTESIS@RISE—Centre for Health Technologies and Services Research, University of Porto, 4200-319 Porto, Portugal; MEDCIDS—Faculty of Medicine of University of Porto, 4200-319 Porto, Portugal; Health Data Science PhD Program, Faculty of Medicine of the University of Porto, 4200-319 Porto, Portugal; CINTESIS@RISE—Centre for Health Technologies and Services Research, University of Porto, 4200-319 Porto, Portugal; MEDCIDS—Faculty of Medicine of University of Porto, 4200-319 Porto, Portugal; Health Data Science PhD Program, Faculty of Medicine of the University of Porto, 4200-319 Porto, Portugal

**Keywords:** data quality, machine-learning, FHIR, real-world data, Bayesian networks

## Abstract

**Background:**

The increasing prevalence of electronic health records (EHRs) in healthcare systems globally has underscored the importance of data quality for clinical decision-making and research, particularly in obstetrics. High-quality data is vital for an accurate representation of patient populations and to avoid erroneous healthcare decisions. However, existing studies have highlighted significant challenges in EHR data quality, necessitating innovative tools and methodologies for effective data quality assessment and improvement.

**Objective:**

This article addresses the critical need for data quality evaluation in obstetrics by developing a novel tool. The tool utilizes Health Level 7 (HL7) Fast Healthcare Interoperable Resources (FHIR) standards in conjunction with Bayesian Networks and expert rules, offering a novel approach to assessing data quality in real-world obstetrics data.

**Methods:**

A harmonized framework focusing on completeness, plausibility, and conformance underpins our methodology. We employed Bayesian networks for advanced probabilistic modeling, integrated outlier detection methods, and a rule-based system grounded in domain-specific knowledge. The development and validation of the tool were based on obstetrics data from 9 Portuguese hospitals, spanning the years 2019-2020.

**Results:**

The developed tool demonstrated strong potential for identifying data quality issues in obstetrics EHRs. Bayesian networks used in the tool showed high performance for various features with area under the receiver operating characteristic curve (AUROC) between 75% and 97%. The tool’s infrastructure and interoperable format as a FHIR Application Programming Interface (API) enables a possible deployment of a real-time data quality assessment in obstetrics settings. Our initial assessments show promised, even when compared with physicians’ assessment of real records, the tool can reach AUROC of 88%, depending on the threshold defined.

**Discussion:**

Our results also show that obstetrics clinical records are difficult to assess in terms of quality and assessments like ours could benefit from more categorical approaches of ranking between bad and good quality.

**Conclusion:**

This study contributes significantly to the field of EHR data quality assessment, with a specific focus on obstetrics. The combination of HL7-FHIR interoperability, machine learning techniques, and expert knowledge presents a robust, adaptable solution to the challenges of healthcare data quality. Future research should explore tailored data quality evaluations for different healthcare contexts, as well as further validation of the tool capabilities, enhancing the tool’s utility across diverse medical domains.

## Introduction

With the wide spreading of healthcare information systems across all contexts of healthcare practice, the production of health-related data has followed this incremental behavior. The potential for using these data to create new clinical knowledge and push medicine further is tempting.^1^ However, to correctly use the data stored in electronic health records (EHRs), the quality of the data must be robust enough to sustain the clinical decisions made based on these data. Data quality cannot be understood as a straightforward concept; it is highly dependent on the context in which it is evaluated. The quality thresholds and dimensions required to classify the quality of the data depend on the purpose that we intend to use that very same data.^2^ These uses can be very distinct and have different impacts as well. For one, we can use data to support day-to-day decisions regarding individual patients’ care.^3^ These decisions can include ones based on recorded information to understand a patient’s history, clinical decision support systems based on these data, or even using the data to help support a more macro, public health-oriented decision. Another area is using information for management purposes. The data can be used by management bodies and regulatory authorities to extract metrics regarding the quality of care or reimbursement purposes. Thirdly, data can be used for research purposes, namely observational studies and, more recently, to support clinical trials through real-world evidence analysis.^3–5^ So, all the EHR data-based decisions can only be as good as the data supporting them. Several studies have already warned about the lack of data quality in EHRs and how this can be a significant hurdle to an accurate representation of the population and potentially lead to erroneous healthcare decisions.^6–11^

There are several steps in the data lifecycle that can be prone to error, from data generation, where the data are registered by healthcare professionals, passing by data processing, whether inside healthcare institutions or by software engineers aiming to reuse data, to data interpretation and reuse, where investigators try to interpret the meaning of registered data.^5^ So, with all the data’s possible uses added to the several steps that can introduce errors throughout the data lifecycle, data quality frameworks and sequential implementations can have very distinct approaches and methodologies to assess data quality. Data quality tools for checking data being registered live to support day-to-day decisions will be significantly different from one whose only purpose is to provide quality checks for research purposes. So, methodologies to tackle these issues are necessary for guaranteeing the quality of healthcare practice and the knowledge derived from EHR data.

There is already a significant number of papers trying to define data quality assessment frameworks for EHR data, all of them plausible and recommendable, already described in other papers.^12^ The literature has over 20 different methods, descriptions, and summaries of different frameworks over the years. Some may be highlighted from the review from Weiskopf and Weng,^13^ where 5 data quality concepts were identified over 230 papers: Completeness, Correctness, Concordance, Plausibility, and Currency. The work of Sáez et al^14^ defined a unified set of Data Quality (DQ) dimensions: completeness, consistency, duplicity, correctness, timeliness, spatial stability, contextualization, predictive value, and reliability. Then a review of Bian et al^12^ expanded on the previous ones, categorizing data quality into 14 dimensions and mapping them to the previous most known definitions. These were: currency, correctness, plausibility, completeness, concordance, comparability, conformance, flexibility, relevance, usability, security, information loss, consistency, and interpretability.

Finally, the work of Kahn et al^15^ tried to harmonize data quality assessment frameworks, which simplified all previous concepts into 3 main categories: conformance, completeness, and plausibility, and 2 assessment contexts: verification and validation. Conformance assesses if data values adhere to specified standards and formats. For instance, checking if a data field like “gender” conforms to accepted values such as “M,” “F,” or “U.” Completeness focuses on whether all necessary data values are present. An example would be checking for missing values in a critical data field like “patient ID.” Plausibility evaluates the believability or truthfulness of data values. An example is verifying that the dates in a dataset (like birth date and date of diagnosis) follow a logical order, where the birth date precedes the diagnosis date. Despite all of these comprehensive works, there is still no consensus regarding which one is best or which has taken the lead in usage. Moreover, looking at all of the descriptions related in the literature, a significant portion of concepts are overlapping, and sometimes hard to conceptualize such dimensions in practice.

As for implementations, there are already some available, such as the work from Phan et al^16^ where a tool created by primary care in the Flanders was built to assess completeness and percentage of values within the normal range. The work from Liaw et al^17^ already reviewed some data quality assessment tools, like tools from OHDSI^18^ or TAQIH.^19^ Additionally, we found some others with similar purposes and characteristics like the work presented data dataquieR,^20^ an R language-based package that can assess several data quality dimensions in observational health research data. Also, the work from Razzaghi et al^21^ developed a methodology for assessing data quality in clinical data, taking into account the semantics of data and their meanings within their context. Furthermore, the work from Rajan et al^22^ presented a tool that can assess data quality and characterize health data repositories. Parallel to this, Kapsner et al^23^ created a tool called DQAStats that enables the profiling and quality assessment of the MIRACUM database, being possible to integrate into other databases as well.

Regarding data quality assessment as a whole, the works of Estiri and Murphy,^24^ focused on outlier detection in large-scale data repositories. The works of Sáez et al^25^ focused on the exploration and identification of dataset shifts, contributing to the broad examination and repurposing of large, longitudinal data sets. The works of Garcı’a-de-Léon-Chocano^26–28^ are the only ones focused on obstetrics data but aimed to improve the process of generating high-quality data repositories for research and best practices monitoring. These are similar and complementary works to this one. Finally, the work of Springate et al^29^ focused on the manipulation of EHR data, including data quality assessment, data cleaning, and data extraction. However, these tools are not meant to be used at the production level, assessing data as it is being registered or outputs reports for human consumption and not a quantitative metric for metric comparison. Furthermore, none of these tools had interoperability in mind. Finally, we have not seen, until the moment of this article, any implementation that used machine learning to evaluate the correctness of the value.

In this article, we aim to achieve the following objectives: (1) Identify and Explain Potential Issues in Full Deployment: We aim to enlighten readers on the various challenges and issues that may arise when fully deploying a tool designed for improving data quality in obstetrics. This involves a detailed analysis of potential technical, operational, and ethical concerns. (2) Develop a Single Data Quality Score: We propose the creation of a comprehensive single score for data quality. This score will facilitate the comparison of high-quality and low-quality records within a database, enabling a more standardized and efficient assessment of data quality. (3) Evaluate Tool Performance in Early-Stage Real-World Scenarios: Our objective is to assess how the proposed tool functions in early-stage real-world scenarios. This includes examining its effectiveness in collaboration with obstetricians and identifying practical strategies for improving data quality based on real-world feedback and conditions.

## Materials and methods

### Materials

The data was gathered from 9 different Portuguese hospitals regarding obstetric information: data from the mother, several data points about the fetus and delivery mode. The data are from 2019 to 2020. The software for collecting data was the same in every institution, and the columns were the same, even though the version of each software differed across hospitals. Across the different hospitals, data rows ranged from 2364 to 18 177. The sum of all rows is 73 351 rows. The data dictionary is in Appendix S1. This study received Institutional Review Board approval from all hospitals included in this study with the following references: Centro Hospitalar São João; 08/2021, Centro Hospitalar Baixo Vouga; 12-03-2021, Unidade Local de Saúde de Matosinho; 39/CES/JAS, Hospital da Senhora da Oliveira; 85/2020, Centro Hosptilar Tamega Sousa; 43/2020, Centro Hospitalar Vila Nova de Gaia/Espinho; 192/2020, Centro Hospitalar entre Douro e Vouga; CA-371/2020-0t_MP/CC, Unidade Local de saúde do Alto Minho; 11/2021. All methods were carried out in accordance with relevant guidelines and regulations. Data was anonymized before usage.

For this purpose, we took the Kahn harmonized framework since we understood it as simpler to communicate we feel that the 3 main categories are indeed non-reducible, which makes sense from an organizational standpoint. Furthermore, the work done by Kahn et al with mapping to already existing frameworks could help compare this work with others who felt the need to use other frameworks. With this in mind, we will use 3 main categories, completeness, plausibility, and conformance. Completeness relates to missing data. Plausibility relates to how believable the values are. Conformance relates to the compliance of the data representation, like formatting, computational conformance, and other data standards implemented.

With this in mind, we will use 3 main categories, Completeness, Conformance, and Plausibility. Completeness relates to missing data. Conformance relates to the compliance of the data representation, like formatting, computational conformance, and other data standards implemented. Plausibility relates to how believable the values are.

### Methods

For completeness, we used the inverse of the percentage of nulls in the training set. For plausibility, several methods were applied. The first was a Bayesian network.

In our approach, Bayesian networks, which are probabilistic graphical models, play a pivotal role in predicting the plausibility of different elements. These networks are structured as directed acyclic graphs, where each node represents a variable and edges denote conditional dependencies among these variables.^30^ This structure allows the network to efficiently manage and represent the probabilistic relationships between multiple variables. The core strength of Bayesian networks in our context lies in their ability to predict the plausibility of various elements by analyzing these interdependencies. By integrating the conditional probabilities of variables and their dependencies, the network can infer the likelihood of certain outcomes or states, thereby assessing the plausibility of different columns in our dataset, when compared with the registered value.

With this, we hope to capture the heterogeneous essence of the data, as well as possible outliers that are also plausible. We chose this model for its dual advantages: its capability to classify the plausibility of all columns within a single unified framework, and its interpretability, which allows for a clearer understanding of how each variable influences the overall plausibility prediction. The networks were created with the pgmpy package.^31^

Secondly, we added the outlier-tree method^32^ which tries to integrate a decision tree that “predicts” the values of each column based on the values of each other column. In the process, every time separation is evaluated, it takes observations from each branch as a homogeneous cluster to search for outliers in the predicted 1-d distribution of the column. Outliers are determined according to confidence intervals in this 1-d distribution and need to have large gaps in order to be marked as outliers in the next observation. Because it looks for outliers in the branch of the decision tree, it knows the conditions that make it a rare observation relative to other observation types corresponding to the same conditions, and these conditions are always related to target variables (as predicted by them). As such, it can only detect outliers described by decision tree logic, and unlike other methods such as isolation forests, it cannot assign outlier points to each observation, or detect outliers that are generally rare, but will always provide human-readable justification when it recognizes outliers. Therefore, these methods not only identify anomalies based on a single column/variable but also consider the context of the data, providing a more nuanced understanding of what constitutes an outlier. This contextual awareness ensures that the outliers are not merely statistical deviations but are also substantively significant within the specific framework of the target variables.

We added also elliptic envelope and Local Outlier Factor as complementary models to these 2. Elliptic envelope is a method that assumes a Gaussian distribution of data, fitting an ellipse to the central data points to identify outliers. It works best with normally distributed data but is less effective in higher dimensions or non-normal distributions. Local Outlier Factor measures the local density deviation of a data point relative to its neighbors, identifying outliers without assuming a specific data distribution. It is versatile for different data structures but sensitive to parameter settings, like the number of neighbors.

An Interquartile Range (IQR) based metric was also added as a supportive metric. This metric used the difference between Q1 and the triple of IQR to define a lower threshold and Q3 + 3IQR to define an upper threshold. We only categorized as outlier the values that fell outside these margins. Finally, a rule system was implemented to leverage domain knowledge in the overall scoring. The system is based on great expectations package.^33^ A set of 17 rules was defined by the team, focusing on impossible numbers or relationship between variables or value format. The rules covered plausibility and conformance.

The Conformance-based were related to technical issues like the format of dates (date of birth like d/m/y), and conformance to the value set (ie, Robson group, bishop scores, or delivery types). Plausibility rules were linked to expected values for BMI, weight, and gestational age (gestational age between 20 and 44). We also added plausibility for the relationship between columns, namely weight across different weeks of gestation (weight week 35 > weight week 25). We have also added a relationship of greatness between ultrasound weights more than 5 weeks apart.

As for preprocessing, all null representations were standardized, we also removed features with high missing rates (>80%). The imputation process was performed with the median for continuous and a new category (NULLIMP) for categorical variables.

For the usage of the Bayesian network in particular, the continuous variables were discretized into 3 bins defined by quantile. We defined 3 as the number of bins in order to reduce the number of states in each node of the network. The evaluation was done with cross-validation with 10 splits and 2 repetitions for each column as the target.

**Figure 1.**
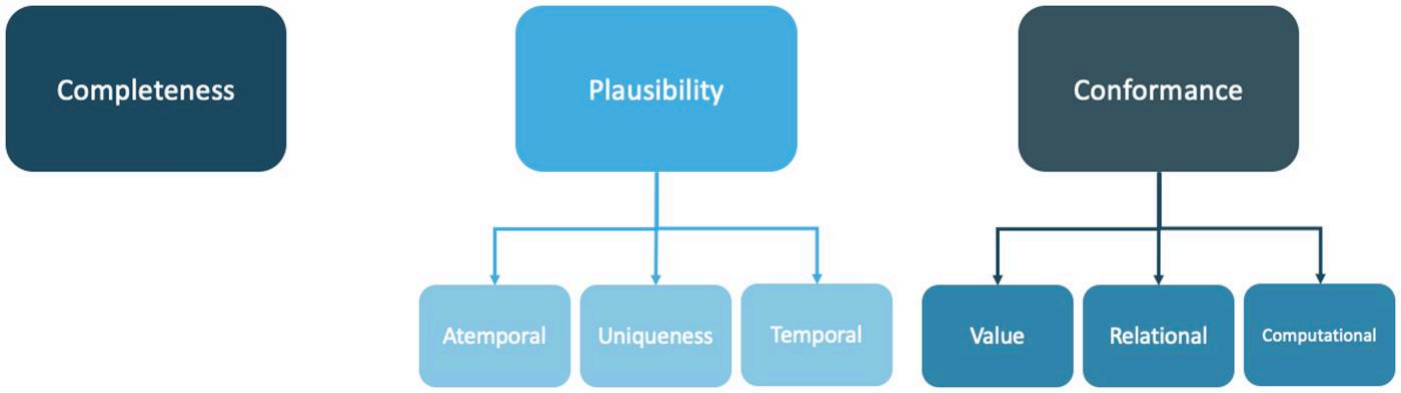
Dimensions of data quality.

The Application Programming Interface (API) for serving the prediction models was developed with FastAPI. So, the methods applied in terms of the DQA framework shown in Figure 1 are described in the Table 1.

**Table 1.** Implemented methods in the tool. The first column is the category or data quality dimension. The second is a subcategory of the first column if applicable and the third column is the actual method used to assess such a dimension.

Category	Subcategory	Method
Completeness	N/A	Score by the inverse percentage of missing in the train data
Plausibility	Atemporal plausibility	Bayesian model prediction based on the other values of row
Plausibility	Atemporal plausibility	*Z*-score for column value based on IQR train data
Plausibility	Atemporal plausibility	Elliptic envelope
Plausibility	Atemporal plausibility	Local outlier factor
Conformance	Value conformance	Manual rule engine
Plausibility	Atemporal plausibility	Manual rule engine
Plausibility	Atemporal plausibility	Outlier-tree
Conformance	Value conformance	Manual rule engine

**Figure 2.**
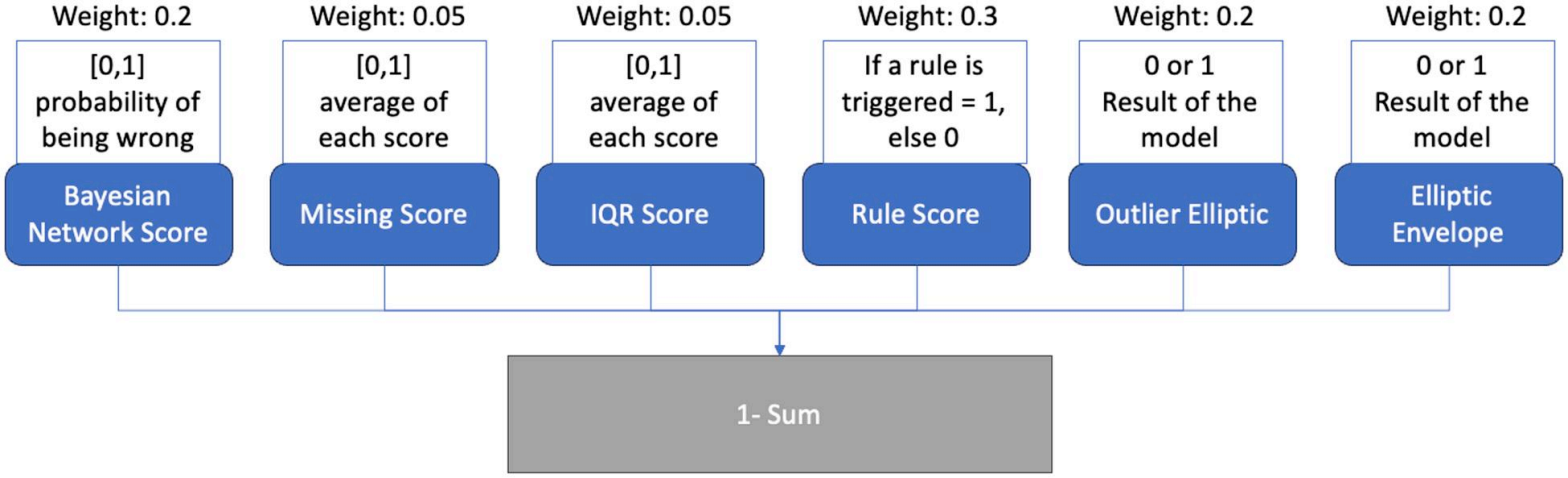
Workflow and weights used for creating the final score and which elements are used to do so.

For trying to compile all of these models into a single value, that could grasp the quality of the row or patient, a scoring method was created. The method of calculating the final score is stated in Figure 2.

To conduct an initial validation of the tool and assess its usefulness, we implemented it in a production environment and collected metrics regarding the data being produced. We then presented selected rows (or patient records) to obstetric clinicians, asking them to assess the likelihood that the information was suitable for use and to rank it according to the perceived quality of the record. This was done through a questionnaire, where clinicians ranked every record from 1 to 10 (one being the best quality one and 10 the worst quality record) and described the most important feature influencing their decision. We then compared the clinicians’ rankings with the model’s results to perform sanity checks on the model’s performance and adequacy.

Firstly, we used Kendall’s Tau and the Average Spearman’s Rank Correlation Coefficient. Kendall’s Tau is a non-parametric statistic that measures the strength and direction of the association between 2 ordinal variables, normalizing the difference between the number of concordant and discordant pairs of observations to ensure a value between −1 (perfect disagreement) and 1 (perfect agreement). Spearman’s rank correlation coefficient is a non-parametric measure that assesses the strength and direction of a monotonic relationship between 2 ranked variables, producing a value between −1 (perfect inverse relationship) and 1 (perfect direct relationship).

Secondly, we used several thresholds to distinguish bad quality records from good quality records, transforming this into a classification problem. We assessed the area under the receiver operating characteristic curve (AUROC) for the model, taking into account the different thresholds. All the code was written in Python 3.10.6, using the scikit-learn library for preprocessing and initial validation.^34^

## Results

**Figure 3.**
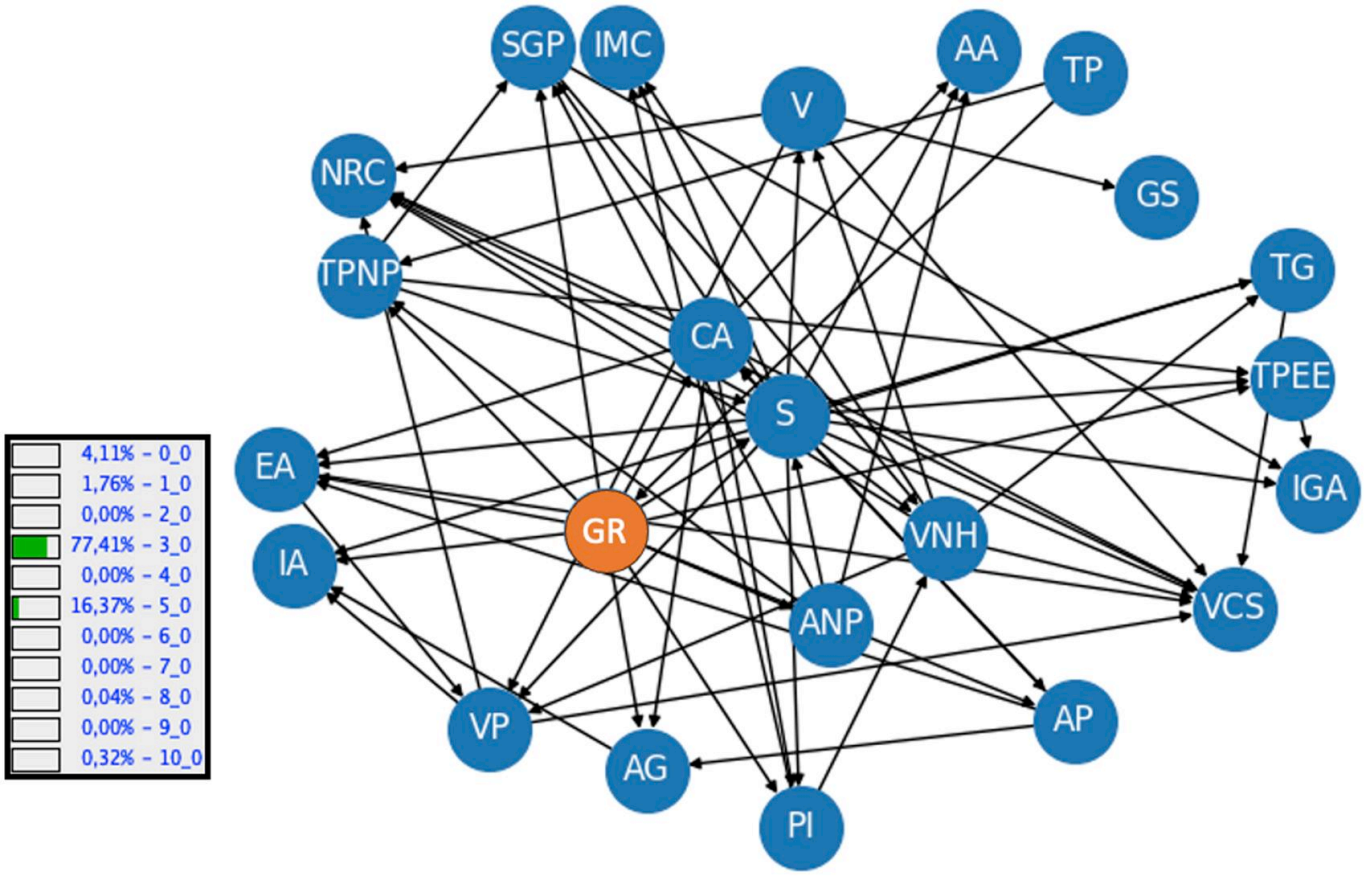
Bayesian Network learned. Nodes acronyms are explained in Appendix S1. The example shows the inference for the Robson Group (10 categories) and the probability of each category, given a set of other features.

Our main result is the tool we developed that we are going to further explore its components. The main one is the Bayesian network developed, and its structure is presented in Figure 3. The example in the image shows the probability for all the classes in the category of Robson group, taking into account the values of the other categories (both known and unknown). In this case, the probability for Robson group number 3 is 77.41%.

The results of the cross validation can be seen in the Table 2. The average AUROC was 0.857. In parentheses is the number of non-null rows that were used in the validation for that column as target.

**Table 2.** Repeated cross-validation (10 × 2) results: column description with area under the receiver operating characteristic curve along with 95% CI. (*n*) is the number of non-null rows.

Name of variable (*n*)	Average	95% CI
Nr of previously born babies (44 387)	0.944	[0.943-0.945]
Nr pregancies (73 335)	0.797	[0.778-0.816]
Nr eutotic deliveries (28 809)	0.969	[0.968-0.969]
Nr prev. C-section (17 879)	0.958	[0.958-0.958]
Mother’s age (73 337)	0.638	[0.637-0.638]
Mother’s weight start (63 324)	0.881	[0.88-0.881]
BMI (62 260)	0.881	[0.881-0.882]
Nr prenatal consultations (61 388)	0.75	[0.75-0.75]
Nr weeks on admission (72 715)	0.968	[0.968-0.969]
Pregnancy weeks on delivery (73 217)	0.974	[0.974-0.974]
Nr deliveries with vacuum (15 985)	0.974	[0.974-0.974]
Pregnancy type (64 517)	0.728	[0.726-0.73]
If pregnancy was accompanied in the hospital (49 738)	0.894	[0.893-0.895]
If delivery was spontaneous (26 360)	0.816	[0.815-0.816]
Baby’s position admission (20 166)	0.751	[0.743-0.758]
Robson group (69 280)	0.931	[0.93-0.932]
If pregnancy was accompanied (73 219)	0.983	[0.982-0.983]
Delivery type (73 350)	0.866	[0.865-0.868]
If was accompanied in the primary care setting (49 812)	0.79	[0.789-0.791]
Baby’s position delivery (73 227)	0.942	[0.938-0.946]
Blood group (73 132)	0.514	[0.507-0.52]
Hospital ID (73 352)	0.896	[0.896-0.897]
If accompanied in a private care setting (18 049)	0.771	[0.77-0.772]
Actual type of delivery (65 606)	0.952	[0.951-0.952]
Average 0.857 [0.846-0.868]		

### Deployment and preliminary validation

The purpose of this model is to serve as an API for usage within a healthcare institution and act as a supplementary data quality assessment tool. Although a concrete, vendor-specific information model and health information system were initially used, our goal is to develop a more universal clinical decision support system. This system should be usable across all systems involved in birth and obstetrics departments. Therefore, we constructed it using the Health Level 7 (HL7) Fast Healthcare Interoperable Resources (FHIR) R5 version standard. This approach simplifies the process of API interaction. Rather than utilizing a proprietary model for the data, we based our decision on the use of FHIR resources: Bundle and Observation. These resources handle the request and response through a customized operation named “$quality_check.” We intend to publish the profiles of these objects to streamline API access via standardized mechanisms and data models. The model then makes use of the customized operation and of several base resources to construct a FHIR message, which are: Bundle, MessageHeader, Observation, Device. Observation is where the information about the record is contained, Device contains information about the model, and MessageHeader is used to add information about the request. Finally, the Bundle is used to group all these resources together. The current version of the profiles can be accessed here.^35^

**Figure 4.**
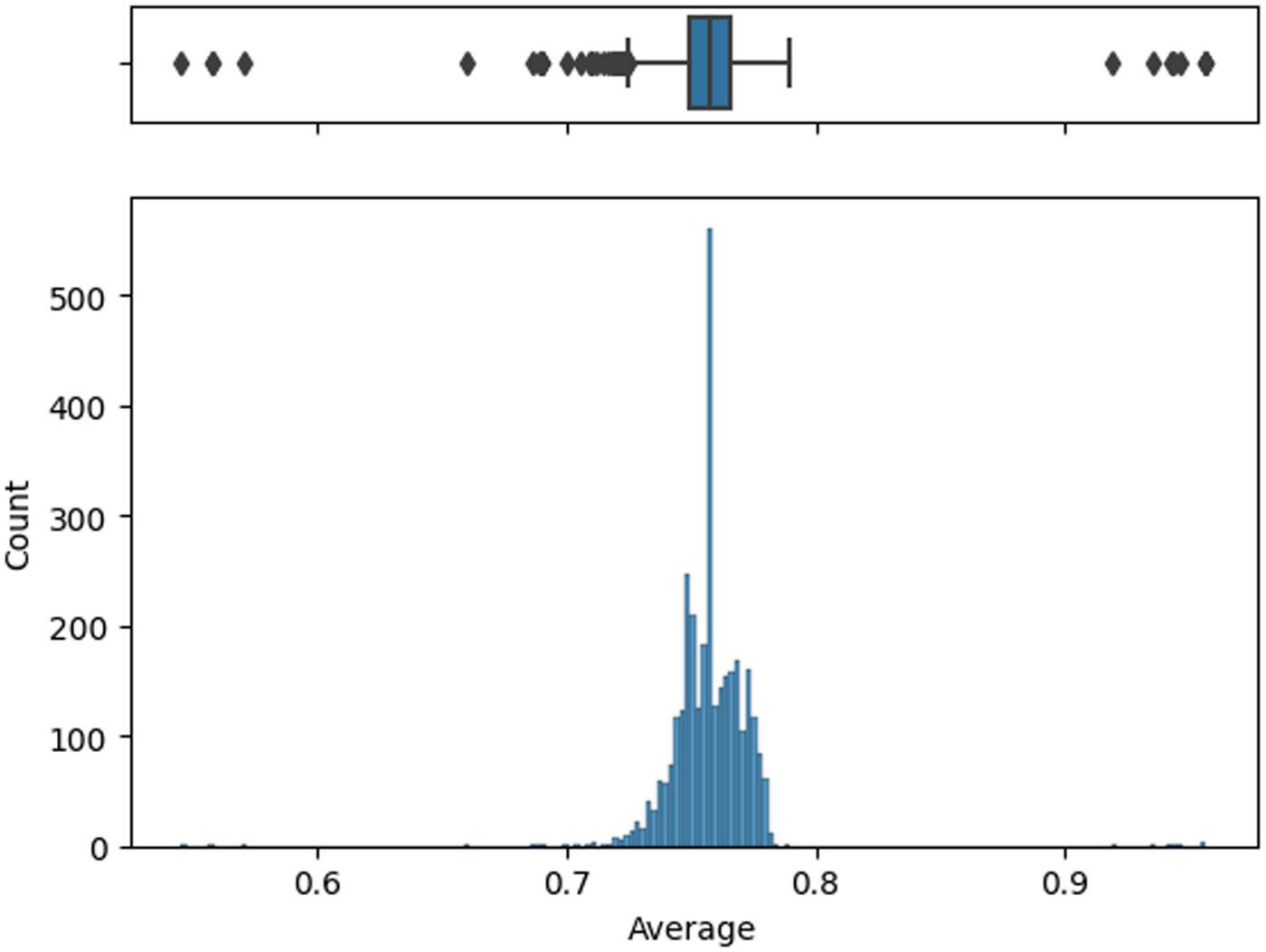
Model score for newly seen data.

**Figure 5.**
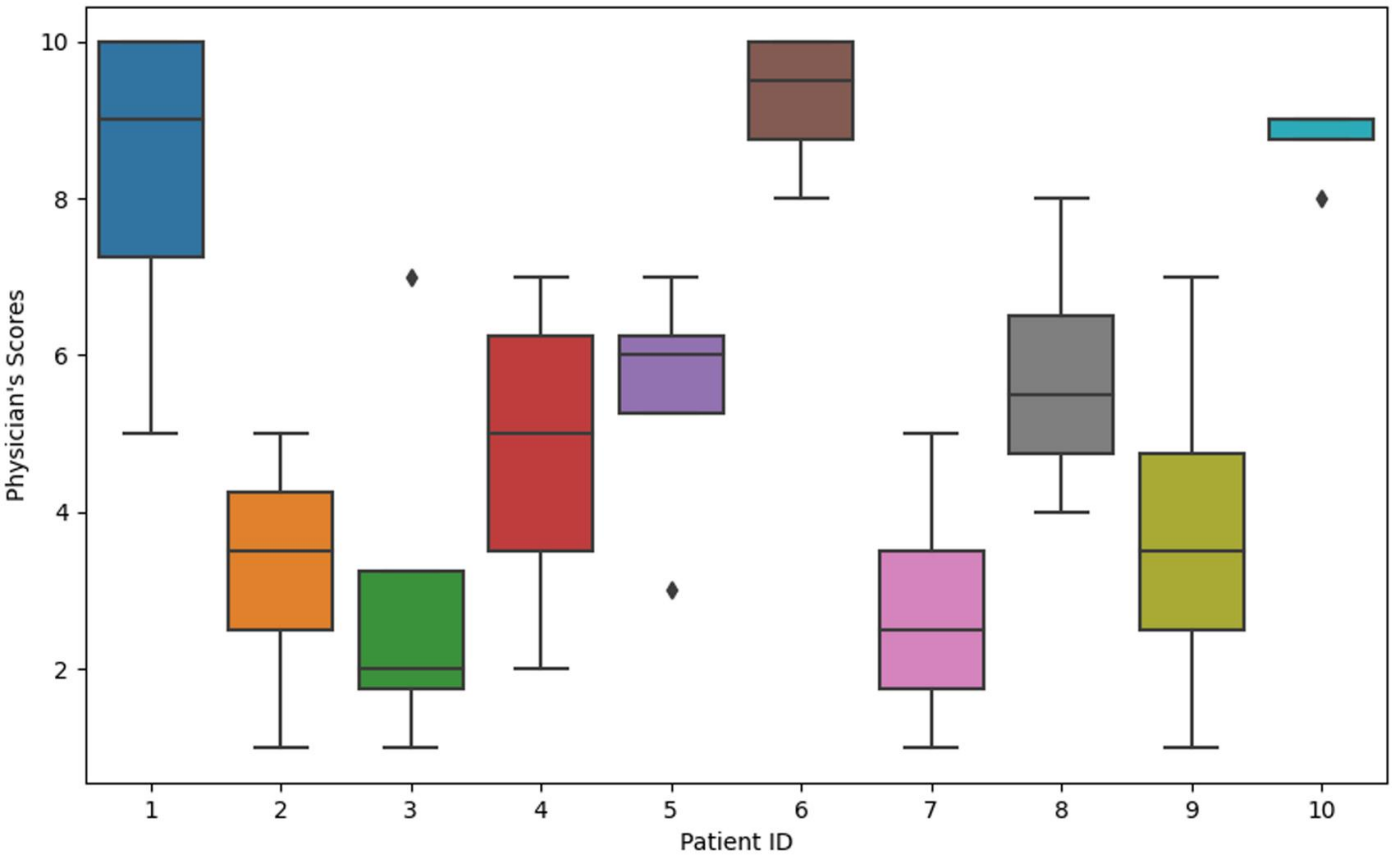
Distribution of rankings obtained from the assessment of 10 records by 4 different clinicians. Y is the distribution of clinicians’ assessment, X is the patient ID.

We conducted a preliminary validation of the tool to assess its initial performance and gather early insights, although a formal, comprehensive assessment was not performed at this stage. In order to do so, we deployed the tool in docker format in a hospital to gather new data. We gathered 3223 new cases and returned a score for quality as exemplified in Figure 4. Being that the score is from 0 to 1, the average score was 0.75 and IQR was 0.016. The formula gives weights to different dimensions since we feel some are more robust than others. We gave more weight to rule system and gave less to the missing and IQR score. Another component of this initial validation was to gather clinicians’ evaluation of random data points from the real-world deployment and compare them with the tool’s assessment. We got 4 answers. Figure 5 shows the distribution of the perceived quality of each record.

**Figure 6.**
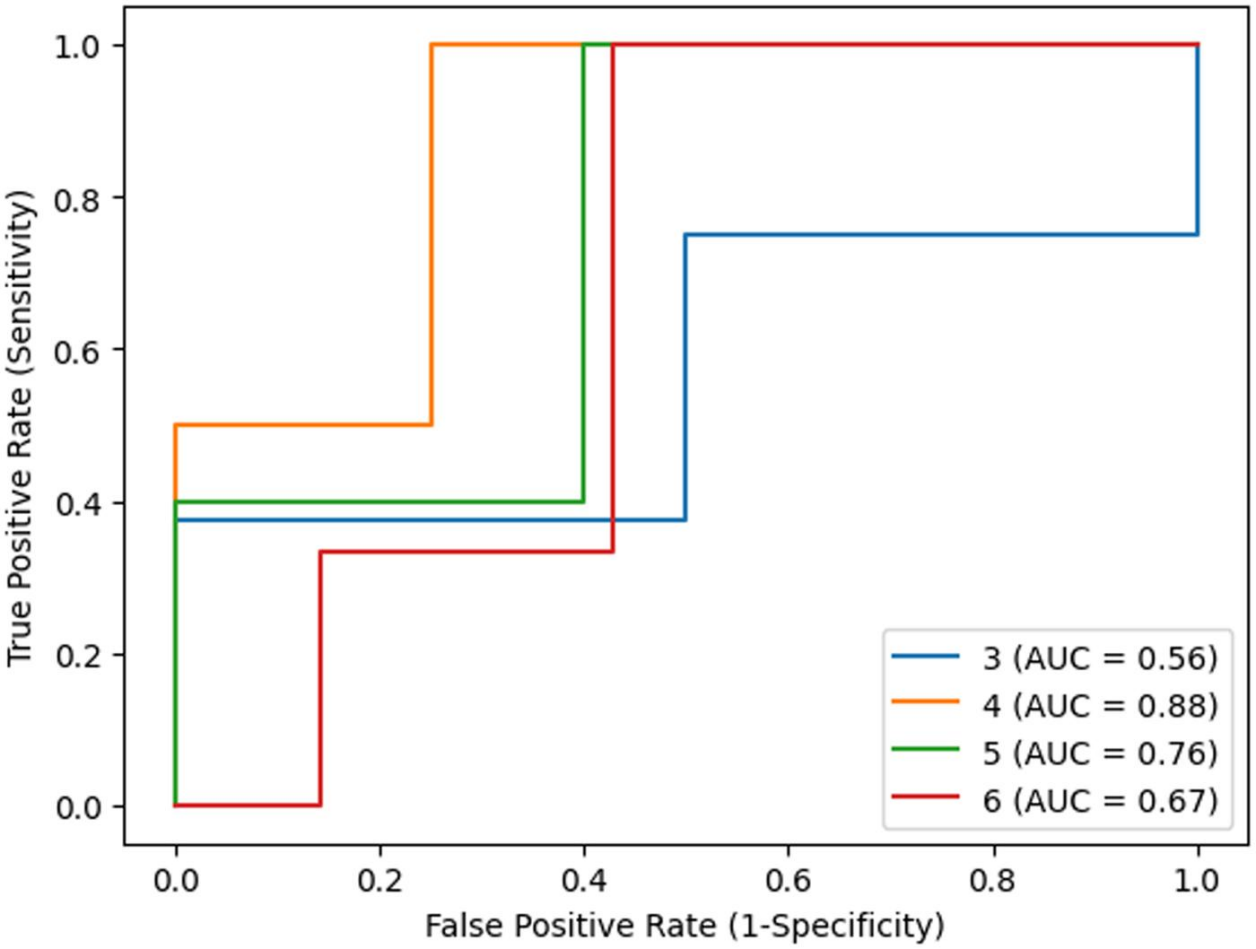
Model Performance in terms of area under the receiver operating characteristic curve (AUROC), depending on the threshold defined on the physician assessed data. The colors show different threshold used to consider a bad quality record given the average ranking. Label shows the threshold and respective AUROC.

Figure 6 shows the performance of the model with several ranking thresholds to differentiate bad quality record from good quality record. Each line/color is a threshold^3^^,^^4^^,^^5^^,^^6^ and the AUC is shown in the label. The Average Spearman’s Rank Correlation Coefficient was 0.42 (*P*-value: .23) and the Kendall’s Tau was 0.3 (*P*-value: .2). Both tests were based on a α of 0.05.

## Discussion

This work adds several pieces of information to the state of the art of data quality analysis. First, we tried to map the output of an automatic assessment tool to the human perception of quality and the issues linked to doing so. Secondly, the fact that we applied explainable machine learning methods such as Bayesian networks to leverage the potency of advanced data analysis without compromising interpretability and explainability. Furthermore, a single model was able to reach high performance metrics for almost all variables. Thirdly, the fact that interoperability standard such as FHIR can be adopted to facilitate the usage and information exchange of such tools. However, there are also shortcoming and challenges to address. The first is that data quality is still an elusive concept since it has a contextual dimension and the quality of the record depends on the usage of the information. For example, data aimed at primary usage and day-to-day healthcare decisions about a patient will have different requirements regarding the importance of some variable or completeness of information very different from data needed to create summary statistics for key performance indicators extraction. Moreover, the data are still very vendor specific. Even though we used an interoperability standard, the semantic layer, more connected with terminology is still lacking. This is an issue to be addressed in order to improve the interoperability of the standard. Moreover, we do not know how the training done with these data are generalizable to other vendors. One opportunity arises of mapping all of these data to a widely used terminology like SNOMED CT or LOINC. Nevertheless, the usage of FHIR and the fact that the data are mapped to a standard terminology, makes it easier to use the data in other systems and to compare the results with other studies. Furthermore, being available freely and online makes it easier to understand how to map vendor-specific datasets to the model and use it in other contexts. Regarding the model, the usage of explainable methodologies like outlier-tree and transparent models like Bayesian networks are vital for clinical application. Since we use a single model to classify possible errors in the records, the ability to try to show clinicians why that value was tagged is of uttermost importance in order to get feedback and action from humans. From the experience gathered with the study, we believe that a weaker but transparent model could have more impact than better performant but opaque ones. If explainability and interpretability are important for any ML problem, this need only increases when we are dealing with such subjective concepts as data quality.

In terms of domain-specific issues, particularly in obstetrics, we found that assessing the quality of a record in an EHR is not an easy task for clinicians. We discovered that for a proper assessment, a context and objective must be defined in order to make the evaluation more objective and manageable. Moreover, the ranking methodology, though very useful for comparison with the model, presents challenges for clinicians who find it difficult to order 10 records when some appear to be of equal quality. This is a very important aspect to consider when designing an evaluation method for data quality. Perhaps a categorical evaluation of yes/no would be more effective than ordering several records. These reasons might explain the great variability between clinicians (Figure 5) and between clinicians and the model (Spearman and Kendall tau). Despite that, our preliminary results are promising, demonstrating an AUROC curve for categorizing bad quality records as high as 88% and low as 56%. The highest value was achieved by classifying all record with a mean rank of 4 or above as bad quality and the others as good quality records. However, these results rely on very few samples, so more data and research are needed in this area since it is a very subjective decision, and it should take into account the context and the objective of the evaluation. For example, if the objective is research use, the weights given to each dimension can be a set. On the other hand, if the objective is to use the data for day-to-day clinical decisions, another set of weights could be used.

For the next steps, a promising research direction would be identifying contexts for applying data quality checks like primary usage, research purposes, and aggregated analysis for decision-making among others. This could enhance targeting those contexts and understanding the importance of each variable for those use cases. Incorporating this approach into the tool to weigh the different variables according to the context would be beneficial. Finally, gaining access to more data and clinician evaluation of records, although challenging, is important to thoroughly assess the performance of the tool.

## Conclusion

We believe the work done is already a valuable insight into how to use data quality frameworks and several statistical tools in order to assess EHR data quality in real time. This is a fundamental process not only to guarantee the quality of data for primary usage but also for securing quality for secondary analysis and usage. We believe the fact that we created an interoperable tool that was trained on real obstetrics data from 9 different hospitals and has the ability to provide a single score for a clinical record can help institutions, academics, and EHR vendors implement data quality assessment tools in their own systems and institutions. With the further evaluation of the score and its relationship with clinical usefulness and a further assessment of a threshold for the score for defining a record that would require human attention would be vital to apply this tool in production with high levels of trust and quality.

## Supplementary Material

ooae062_Supplementary_Data

## Data Availability

The data underlying this article were provided by the hospital by permission. Data will be shared on request to the corresponding author with permission of the hospitals.
