# Supplementary material for: Development and initial validation of a data quality evaluation tool in obstetrics real-world data through HL7-FHIR interoperable Bayesian networks and expert rules
Source: JAMIA Open. 2024 Jul 27;7(3):ooae062. doi: 10.1093/jamiaopen/ooae062 (PMC11283181; doi:10.1093/jamiaopen/ooae062)
Supplement: ooae062_Supplementary_Data [file ooae062_supplementary_data.docx]

# Data Dictionary

| Initial | Description |
| --- | --- |
| IA | Mother Age |
| GS | Blood Group |
| PI | Weight at the beginning of pregnancy |
| PAI | Weight on Admission |
| IMC | BMI |
| CIG | If Smoker During Pregnancy |
| APARA | Number of previously born babies |
| AGESTA | Number of Pregnancies |
| EA | Number of Previous Eutocic Deliveries with no assistance |
| VA | Number of Previous Eutocic Deliveries with help of vacuum extraction |
| FA | Number of Previous Eutocic Deliveries with help of forceps |
| CA | Number of Previous C-sections |
| TG | Pregnancy Type (spontaneous, In vitro fertilisation...) |
| V | If the pregnancy was accompanied by physician |
| NRCPN | Number of prenatal consultations |
| VH | If the pregnancy was accompanied by a physician in a hospital |
| VP | If the pregnancy was accompanied by a physician in a private clinic |
| VCS | If the pregnancy was accompanied by a physician in a primary care facility |
| VNH | If the pregnancy was accompanied by a physician in the hospital the delivery was made |
| B | Pelvis Adequacy |
| AA | Baby’s Position on Admission |
| BS | Bishop Score |
| BC | Bishop Score Cervical Consistency |
| BDE | Bishop Score Fetal Station |
| BDI | Bishop Score Dilatation |
| BE | Bishop Score Effacement |
| BP | Bishop Score Cervical Position |
| IGA | Number of Weeks on Admission |
| TPEE | If the delivery was spontaneous |
| TPEI | If the delivery was induced |
| RPM | If there was a rupture of the amniotic pocket before delivery began |
| DG | Gestational Diabetes |
| TP | Delivery Type |
| ANP | Baby’s Position on Delivery |
| TPNP | Actual Type of Delivery |
| SGP | Pregnancy Weeks on Delivery |
| GR | Robson Group |
